# Supplementary material for: From sequence to enzyme mechanism using multi-label machine learning
Source: BMC Bioinformatics. 2014 May 19;15:150. doi: 10.1186/1471-2105-15-150 (PMC4229970; doi:10.1186/1471-2105-15-150)
Supplement: Additional file 2 — Java code of ml2db. Additional file ml2db_code.tar.gz contains the Java source code to run the multi-label machine learning experiments and save the results to database. The code’s Javadoc is included. [file 1471-2105-15-150-S2.zip › additional file 2/ml2db/ml2db/doc/mulan/data/LabelsMetaDataImpl.html]

LabelsMetaDataImpl


JavaScript is disabled on your browser.


- Overview
- Package
- Class
- Use
- Tree
- Deprecated
- Index
- Help

- Prev Class
- Next Class

- Frames
- No Frames

- All Classes

- Summary:
- Nested |
- Field |
- Constr |
- Method

- Detail:
- Field |
- Constr |
- Method


mulan.data

## Class LabelsMetaDataImpl

- java.lang.Object
- - mulan.data.LabelsMetaDataImpl

- All Implemented Interfaces:
  :   java.io.Externalizable, java.io.Serializable, LabelsMetaData

  ---

    

  ```
  public class LabelsMetaDataImpl
  extends java.lang.Object
  implements LabelsMetaData, java.io.Serializable, java.io.Externalizable
  ```

  Implementation of `LabelsMetaData` info about labels and their structure.

  Author:
  :   Jozef Vilcek

  See Also:
  :   `LabelsMetaData`,
      Serialized Form

- - ### Constructor Summary

    Constructors

    Constructor and Description

    `LabelsMetaDataImpl()`

    Creates a new instance of `LabelsMetaDataImpl`.
  - ### Method Summary

    Methods

    | Modifier and Type | Method and Description |
    | --- | --- |
    | `void` | `addRootNode(LabelNode rootNode)` Adds a root `LabelNode`. |
    | `LabelsMetaData` | `clone()` Returns a deep copy of the `LabelsMetaDataImpl` instance. |
    | `boolean` | `containsLabel(java.lang.String labelName)` Determines if `LabelsMetaData` contains a label with specified name. |
    | `java.util.Set<java.lang.String>` | `getLabelNames()` Gets the names of all labels. |
    | `LabelNode` | `getLabelNode(java.lang.String labelName)` Gets the `LabelNode` specified by label name. |
    | `int` | `getNumLabels()` Gets the total number of `LabelNode` nodes. |
    | `java.util.Set<LabelNode>` | `getRootLabels()` Gets the unmodifiable `Set` of root `LabelNode` nodes of label attributes hierarchy. |
    | `boolean` | `isHierarchy()` Determines if there is a hierarchy defined between labels. |
    | `void` | `readExternal(java.io.ObjectInput in)` |
    | `int` | `removeLabelNode(java.lang.String labelName)` Removes `LabelNode` specified by the name. |
    | `void` | `writeExternal(java.io.ObjectOutput out)` |

    - ### Methods inherited from class java.lang.Object

      `equals, getClass, hashCode, notify, notifyAll, toString, wait, wait, wait`

- - ### Constructor Detail


    - #### LabelsMetaDataImpl

      ```
      public LabelsMetaDataImpl()
      ```

      Creates a new instance of `LabelsMetaDataImpl`.
  - ### Method Detail


    - #### addRootNode

      ```
      public void addRootNode(LabelNode rootNode)
      ```

      Adds a root `LabelNode`. The node is assumed to has linked all
      related child nodes, if they exists.
      The node is added into root set and all child nodes are added into internal mapping.
      The node is uniquely identified by the label name.

      Parameters:
      :   `rootNode` - the root node with underlying hierarchy of nodes

      Throws:
      :   `ArgumentNullException` - if specified root node is null


    - #### getLabelNode

      ```
      public LabelNode getLabelNode(java.lang.String labelName)
      ```

      **Description copied from interface: `LabelsMetaData`**

      Gets the `LabelNode` specified by label name. The name is unique identifier
      of the node and corresponds to label attribute in the data set.
      If `LabelNode` for given label name does not exists, `null` is returned.

      **Specified by:**
      :   `getLabelNode` in interface `LabelsMetaData`

      Parameters:
      :   `labelName` - the name of label of which the node should be retrieved

      Returns:
      :   the `LabelNode` for specified label of `null`
          if `LabelNode` does not exists for specified label name


    - #### getLabelNames

      ```
      public java.util.Set<java.lang.String> getLabelNames()
      ```

      **Description copied from interface: `LabelsMetaData`**

      Gets the names of all labels. The label name is a unique identifier among all
      labels in the meta data.

      **Specified by:**
      :   `getLabelNames` in interface `LabelsMetaData`

      Returns:
      :   the names of all labels.


    - #### containsLabel

      ```
      public boolean containsLabel(java.lang.String labelName)
      ```

      **Description copied from interface: `LabelsMetaData`**

      Determines if `LabelsMetaData` contains a label with specified name.

      **Specified by:**
      :   `containsLabel` in interface `LabelsMetaData`

      Parameters:
      :   `labelName` - the label name

      Returns:
      :   `true` if meta data contains the given label name;
          `false` otherwise.


    - #### isHierarchy

      ```
      public boolean isHierarchy()
      ```

      **Description copied from interface: `LabelsMetaData`**

      Determines if there is a hierarchy defined between labels. If not, all labels are
      represented as root `LabelNode` nodes.

      **Specified by:**
      :   `isHierarchy` in interface `LabelsMetaData`

      Returns:
      :   `true` if there is hierarchy defined between labels;
          `false` otherwise.


    - #### getNumLabels

      ```
      public int getNumLabels()
      ```

      **Description copied from interface: `LabelsMetaData`**

      Gets the total number of `LabelNode` nodes.

      **Specified by:**
      :   `getNumLabels` in interface `LabelsMetaData`

      Returns:
      :   total number of `LabelNode` nodes


    - #### getRootLabels

      ```
      public java.util.Set<LabelNode> getRootLabels()
      ```

      **Description copied from interface: `LabelsMetaData`**

      Gets the unmodifiable `Set` of root `LabelNode`
      nodes of label attributes hierarchy.

      **Specified by:**
      :   `getRootLabels` in interface `LabelsMetaData`

      Returns:
      :   the `Set` of root nodes.


    - #### clone

      ```
      public LabelsMetaData clone()
      ```

      **Description copied from interface: `LabelsMetaData`**

      Returns a deep copy of the `LabelsMetaDataImpl` instance.

      **Specified by:**
      :   `clone` in interface `LabelsMetaData`

      **Overrides:**
      :   `clone` in class `java.lang.Object`

      Returns:
      :   a deep copy of the `LabelsMetaDataImpl` instance


    - #### removeLabelNode

      ```
      public int removeLabelNode(java.lang.String labelName)
      ```

      Removes `LabelNode` specified by the name. If there is a hierarchy between
      label nodes, whole subtree with all its children is also removed.

      Parameters:
      :   `labelName` - the name of `LabelNode` to be removed

      Returns:
      :   the number of removed nodes


    - #### readExternal

      ```
      public void readExternal(java.io.ObjectInput in)
                        throws java.io.IOException,
                               java.lang.ClassNotFoundException
      ```

      **Specified by:**
      :   `readExternal` in interface `java.io.Externalizable`

      Throws:
      :   `java.io.IOException`
      :   `java.lang.ClassNotFoundException`


    - #### writeExternal

      ```
      public void writeExternal(java.io.ObjectOutput out)
                         throws java.io.IOException
      ```

      **Specified by:**
      :   `writeExternal` in interface `java.io.Externalizable`

      Throws:
      :   `java.io.IOException`


- Overview
- Package
- Class
- Use
- Tree
- Deprecated
- Index
- Help

- Prev Class
- Next Class

- Frames
- No Frames

- All Classes

- Summary:
- Nested |
- Field |
- Constr |
- Method

- Detail:
- Field |
- Constr |
- Method
